# Supplementary material for: A comprehensive genetic map of cytokine responses in Lyme borreliosis
Source: Nat Commun. 2024 May 7;15:3795. doi: 10.1038/s41467-024-47505-z (PMC11076587; doi:10.1038/s41467-024-47505-z)
Supplement: Supplementary file 1 — Supplementary Information [file 41467_2024_47505_MOESM1_ESM.pdf]

	**LB patients (n=1,059)**
Male sex - no (%)	435 (41.1%)
Age (years)	
median [min, max]	55 [19, 87]
mean (SD)	53.5 (12.5)
LB manifestation	
EM	1,017 (96%)
Disseminated LB	42 (4%)
Ancestry	European
Blood taken before the start of antibiotic treatment?	
Yes	
No	128 (13.1%)
	847 (86.9 %)

Supplementary Table 1. Patient characteristics LymeProspect cohort.

Locus Nr.	Lead SNP							Cytokine	Stimulation	Time	Cell System	eQTL	Bb-responding
	SNP	Chromosome	Position	REF	ALT	ALT_AF	P						
1	rs78029196	1	155980434	A	C	0.01599	1.22e-08	IL-10	Bbmix 10-6	After treatment	PBMC	PAQR6, C1orf85	
2	rs10922097	1	196664482	C	T	0.61255	1.74e-08	IL-1ra	C.albicans	Baseline	PBMC	CFHR3, CFH, CFHR1	
3	rs1518111	1	206944645	C	T	0.20901	1.85e-09	IL-10	Bbmix MOI 30	After treatment	whole blood	IL10, IL19, IL24, FAIM3	IL10, IL19, IL24, FAIM3
4	rs184402738	2	174185548	C	G	0.03565	4.08e-08	IL-6	Bbmix MOI 10	Baseline	whole blood		
5	rs72916599	2	192220237	G	T	0.96894	3.43e-08	IL-6	LPS	After treatment	whole blood		
6	rs186427437	3	635601	G	T	0.01048	8.58e-09	IL-6	LPS	Baseline	whole blood		
8	rs62311744	4	107695885	A	G	0.86204	2.05e-08	IL-10	LPS	After treatment	PBMC		
9	rs139808583	4	188881743	A	G	0.01345	3.59e-08	IL-1ra	LPS	Baseline	PBMC		
11	rs189277163	5	78658821	A	G	0.98954	4.48e-09	IL-6	Bbmix MOI 30	After treatment	whole blood		
12	rs76041464	5	125748282	A	C	0.98796	4.54e-08	IL-10	P3C	After treatment	PBMC		
13	rs114773182	6	24081453	A	T	0.98923	7.00e-09	IL-6	LPS	After treatment	whole blood		
14	rs17690267	6	73244359	C	T	0.01050	4.60e-08	IL-6	Bbmix MOI 30	After treatment	whole blood		
15	rs79635669	6	107194632	C	G	0.9436	4.62e-08	IL-6	Bbmix 1e5	After treatment	PBMC	SLC30A5, QRSL1	
17	rs141247920	7	50455346	C	T	0.01050	6.88e-09	IL-1b	Bbmix MOI 10	After treatment	whole blood	SLC38A5, NSUN3, DHFRL1, C2orf88, MYL4, OPTN, RIOK3, BPGM, FIGNL1	
18	rs79354203	8	57116439	A	C	0.98561	5.81e-09	IL-10	Bbmix 10-6	Baseline	PBMC		
20	rs143793226	8	138644913	C	T	0.98968	3.91e-09	IL-1b	Bbmix MOI 10	After treatment	whole blood		
21	rs149459838	9	32597200	A	G	0.01043	3.00e-08	IL-1b	Bbmix MOI 10	After treatment	whole blood		
22	rs76610496	9	81619159	A	G	0.98483	2.68e-09	IL-1ra	LPS, P3C	Baseline	PBMC	LY6G5C	
23	rs117871407	11	46237369	C	T	0.01382	1.50e-08	IL-6	LPS	After treatment	whole blood		
24	rs74501326	11	79108246	A	G	0.05530	3.21e-08	IL-1ra	Bbmix 10-6	After treatment	PBMC	TENM4	
25	rs145399698	13	27774981	C	T	0.01289	1.74e-09	IL-10	LPS	After treatment	whole blood	SIGMAR1	
26	rs525014	13	33599198	A	G	0.71144	3.90e-08	IL-6	Bbmix MOI 10	After treatment	whole blood	KL	KL
28	rs147537541	13	88293742	C	T	0.02575	8.36e-09	IL-6	P3C	After treatment	PBMC		
29	rs72653590	13	111974844	A	G	0.89157	2.09e-08	IL-10	Bbmix 1e5	Baseline	PBMC	ARHGEF7, TEX29, ANKRD10	
30	rs189347006	14	64799698	A	C	0.98492	4.04e-08	IL-1ra	Bbmix 10-4	Baseline	PBMC		
31	rs148048044	18	5538626	A	G	0.98546	3.14e-08	IL-6	Bbmix MOI 30	After treatment	whole blood		
32	rs140993919	19	22148360	C	T	0.02058	8.65e-09	IL-10	C.albicans	After treatment	PBMC	RP11-678G14.3, ZNF429, RP11-678G14.4, CTD-2561J22.4, RP11-420K14.2	CTD-2561J22.4
33	rs141182016	20	1452303	C	T	0.01283	3.33e-08	IL-6	LPS	After treatment	whole blood	SIRPB2, SIRPB1, NSFL1C	SIRPB2,
34	rs1860216	22	30685495	A	G	0.98049	2.51e-08	IL-6	LPS	After treatment	whole blood	NDUFA13, TSSK6, YJEFN3, MTMR3, GATSL3, TCN2, SLC35E4, NEFH	SLC35E4, GATSL3, TCN2, NEFH

Supplementary Table 2. Genome-wide cQTL results. Lead SNP, information on the lead SNP per locus. Cytokine, Stimulation, Time, Cell System, conditions at which the genome-wide association is found. eQTL, lead SNP eQTL effects in whole blood in the eQTLgen summary statistics. DE, differentially-expressed genes upon *B. burgdorferi* stimulation among the eQTL genes. P-values are calculated based on a linear model associating cytokine concentrations with genetic variants and are shown without multiple testing correction. Only p-values below the genomewide significance threshold are shown ($P<5e-8$).

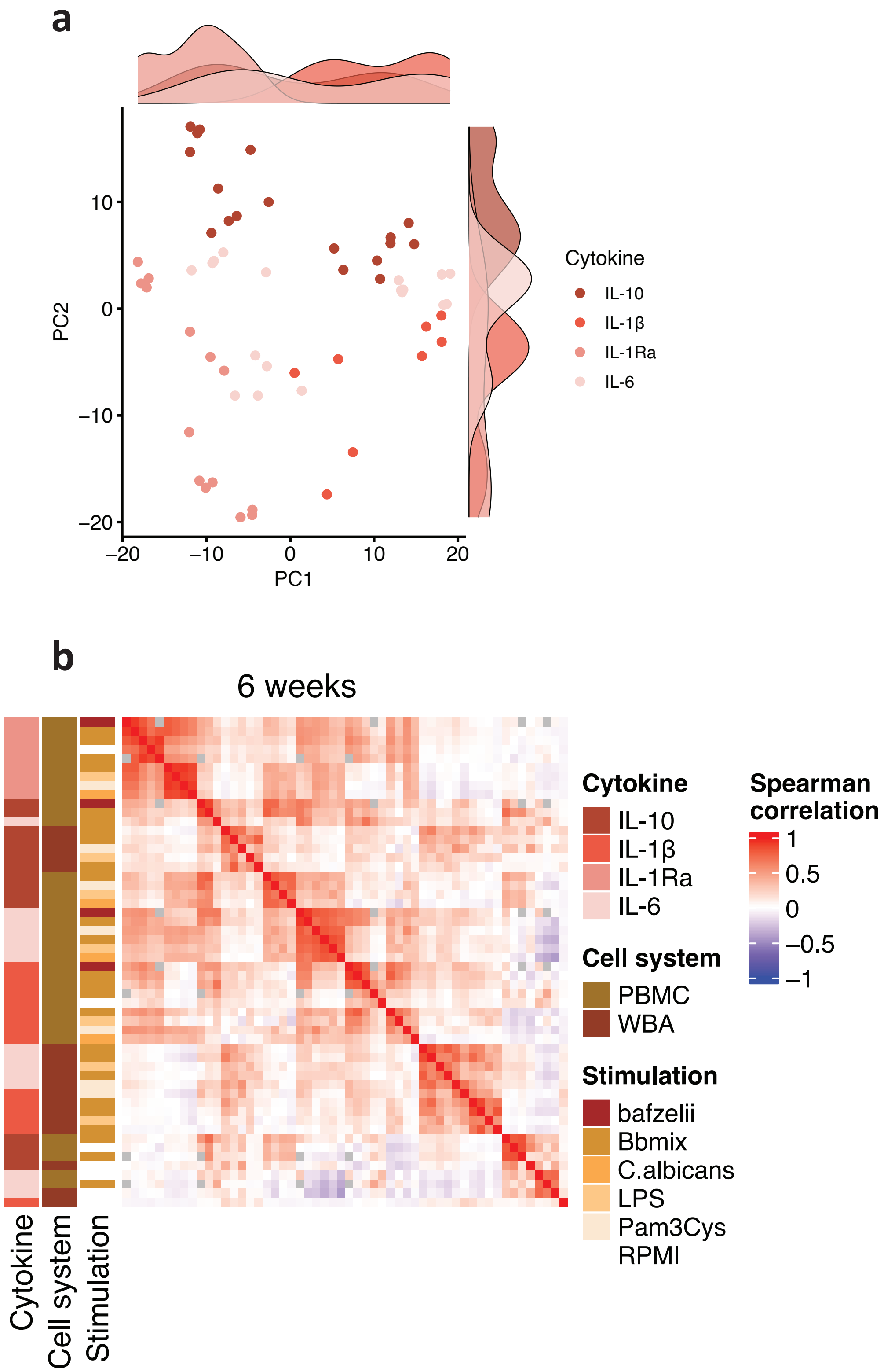
a
PC2
PC1
10
0
−10
−20
20
Cytokine
IL-10
IL-1β
IL-1Ra
IL-6
b
6 weeks
Cytokine
Cell system
Stimulation
Cytokine
IL-10
IL-1β
IL-1Ra
IL-6
Cell system
PBMC
WBA
Stimulation
bafzelii
Bbmix
C.albicans
LPS
Pam3Cys
RPMI
Spearman correlation
1
0.5
0
−0.5
−1

Supplementary Figure 1. a, PCA plot of cytokine-stimulation values. PCA of imputed log2 transformed values, performed on the features used throughout the whole paper, including both time points, PBMCs and whole blood. Dot colour indicates the cytokine measured. b, Heatmap of cytokine-stimulation correlation. Spearman's correlation of the cytokine concentrations. Correlation matrices were split by the time of the sampling, i.e at start of antibiotic treatment (Baseline) or 6 weeks thereafter.

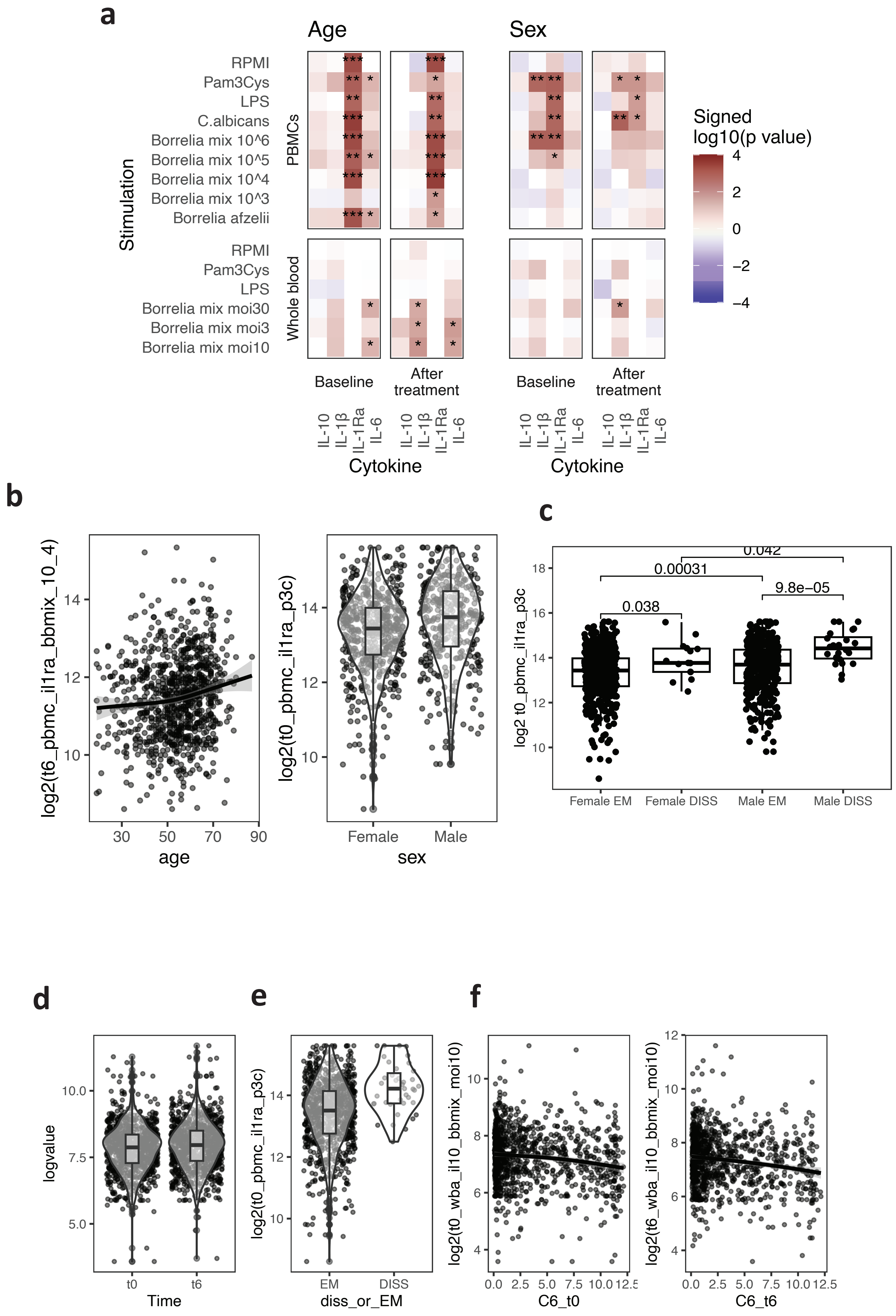

a
Age
Sex
Stimulation
PBMCs
Whole blood
RPMI
Pam3Cys
LPS
C.albicans
Borrelia mix 10^6
Borrelia mix 10^5
Borrelia mix 10^4
Borrelia mix 10^3
Borrelia afzelii
RPMI
Pam3Cys
LPS
Borrelia mix moi30
Borrelia mix moi3
Borrelia mix moi10
Baseline
After treatment
Baseline
After treatment
IL-10
IL-1β
IL-1Ra
IL-6
Cytokine
Cytokine
Signed log10(p value)
4
2
0
−2
−4
b
log2(t6_pbmc_il1ra_bbmix_10_4)
age
30
50
70
90
log2(t0_pbmc_il1ra_p3c)
sex
Female
Male
c
log2 t0_pbmc_il1ra_p3c
0.042
0.00031
0.038
9.8e−05
Female EM
Female DISS
Male EM
Male DISS
d
logvalue
Time
t0
t6
e
log2(t0_pbmc_il1ra_p3c)
diss_or_EM
EM
DISS
f
log2(t0_wba_il10_bbmix_moi10)
C6_t0
log2(t6_wba_il10_bbmix_moi10)
C6_t6

Supplementary Figure 2. a, Heatmap of the significance of associations. * FDR<0.05 ** FDR<0.01 *** FDR<0.001. Age, Spearman's correlation of Age and Cytokine concentrations. Sex, two-sided Wilcoxon rank-sum test between both sexes, red indicates higher in male. b, Exemplification of two significant age correlations and sex differences. c, Sex and disseminated phenotype effects, p-values calculated by Wilcoxon rank-sum test. d,e,f Examples of top associations found in Figure 1c-e. EM, erythema migrans; DISS, disseminated infection. n=1,060.

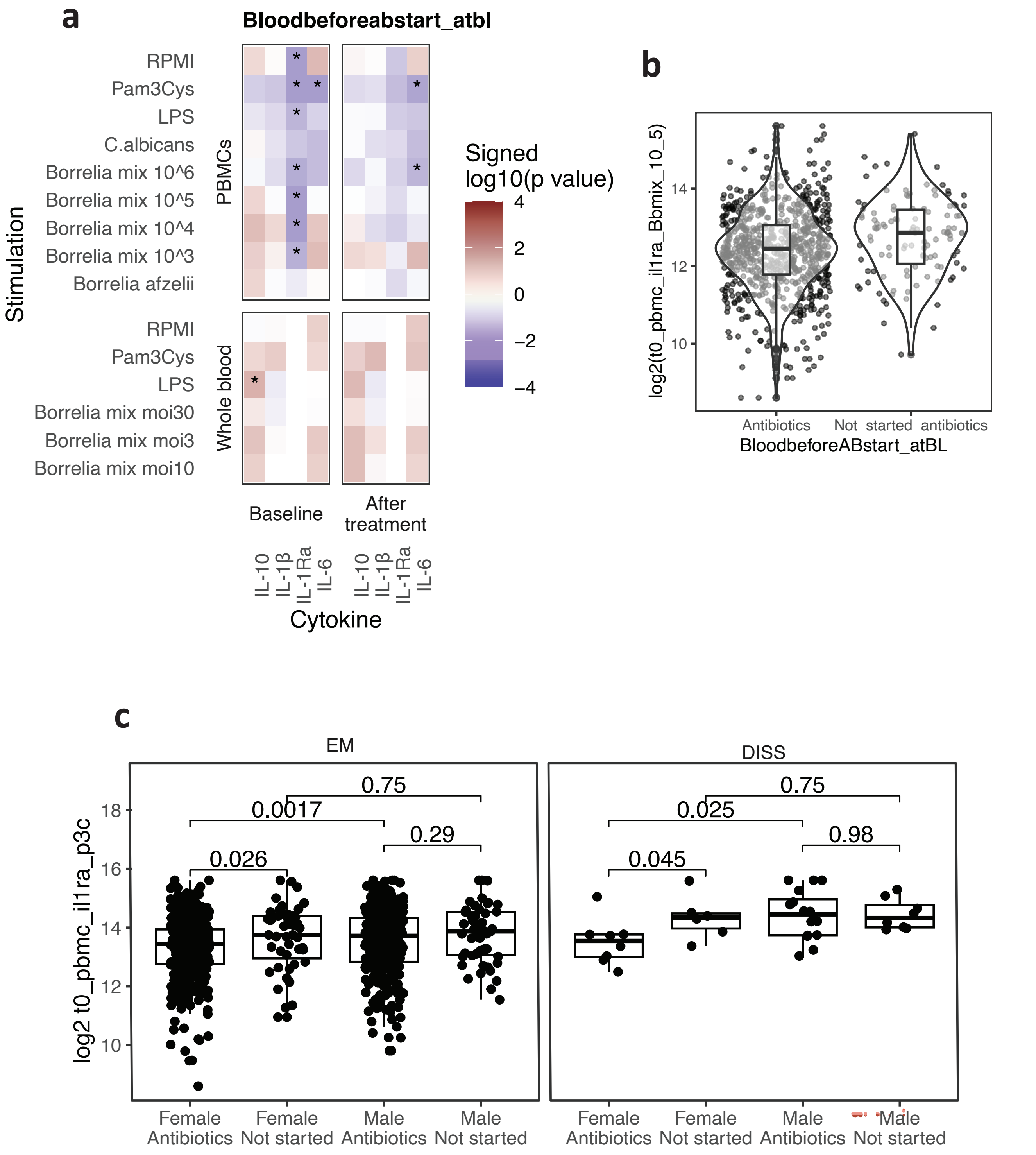
a
Bloodbeforeabstart_atbl
Stimulation
RPMI
Pam3Cys
LPS
C.albicans
Borrelia mix 10^6
Borrelia mix 10^5
Borrelia mix 10^4
Borrelia mix 10^3
Borrelia afzelii
PBMCs
RPMI
Pam3Cys
LPS
Borrelia mix moi30
Borrelia mix moi3
Borrelia mix moi10
Whole blood
Baseline
After treatment
IL-10
IL-1β
IL-1Ra
IL-6
Cytokine
Signed log10(p value)
4
2
0
−2
−4
b
log2(t0_pbmc_il1ra_Bbmix_10_5)
14
12
10
Antibiotics
Not_started_antibiotics
BloodbeforeABstart_atBL
c
EM
DISS
log2 t0_pbmc_il1ra_p3c
18
16
14
12
10
0.75
0.0017
0.29
0.026
0.75
0.025
0.98
0.045
Female Antibiotics
Female Not started
Male Antibiotics
Male Not started

Supplementary Figure 3. Cytokine responses are influenced by the start of antibiotic treatment. a, Heatmap of the significance of associations. * FDR<0.05 ** FDR<0.01 *** FDR<0.001. Two-sided wilcoxon rank-sum test between patients that had and patients that had not started antibiotic treatment at the moment baseline blood samples were taken. Red indicates higher in those that had already started antibiotics. b, Boxplot as an example of the top change with antibiotic start. c, Interaction between antibiotic start, sex and disseminated disease. P-values are calculated by two-sided Wilcoxon rank-sum test. n=1,060.

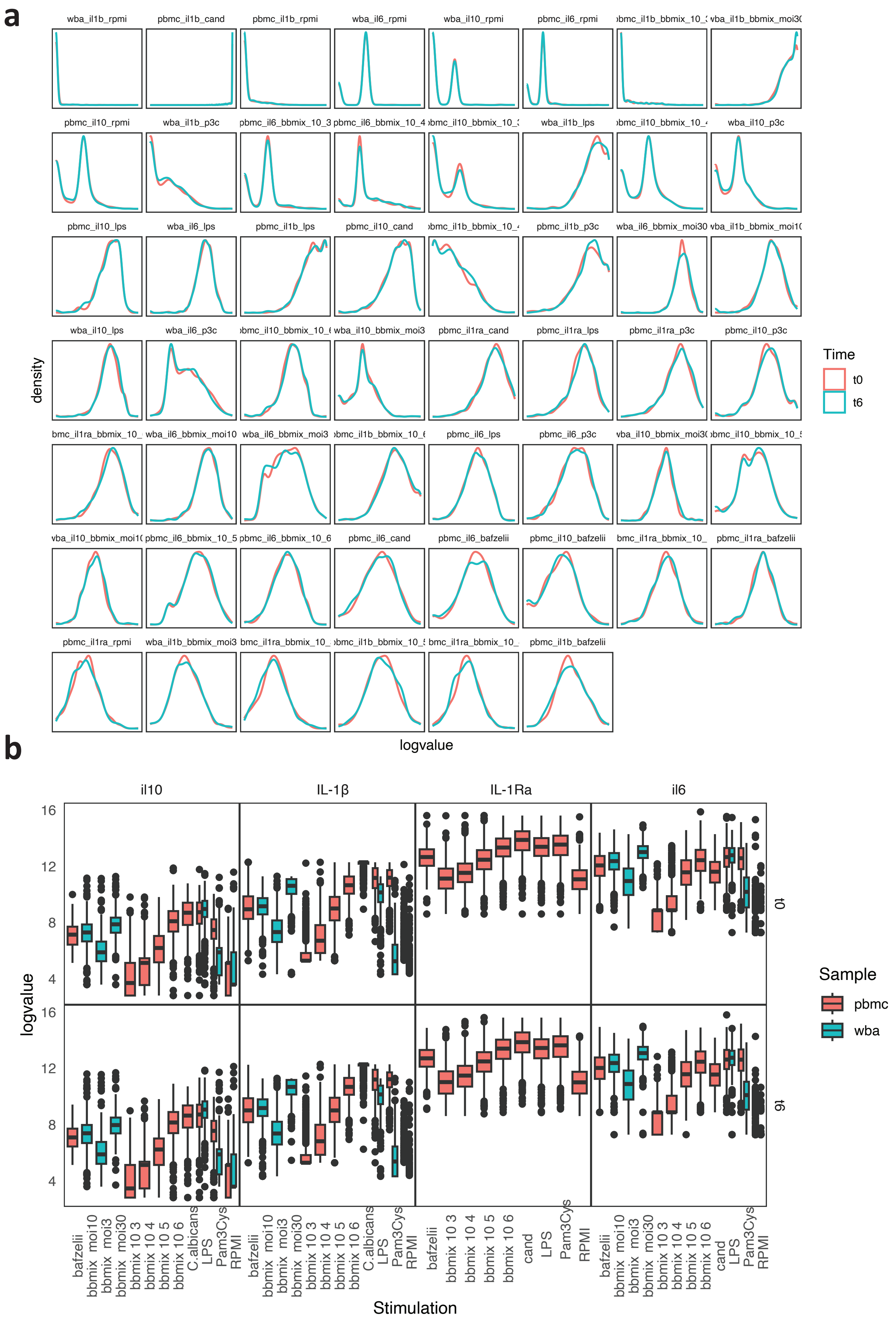
a
wba_il1b_rpmi
pbmc_il1b_cand
pbmc_il1b_rpmi
wba_il6_rpmi
wba_il10_rpmi
pbmc_il6_rpmi
bmc_il1b_bbmix_10_
vba_il1b_bbmix_moi3
pbmc_il10_rpmi
wba_il1b_p3c
pbmc_il6_bbmix_10_3
pbmc_il6_bbmix_10_4
bmc_il10_bbmix_10_
wba_il1b_lps
bmc_il10_bbmix_10_
wba_il10_p3c
pbmc_il10_lps
wba_il6_lps
pbmc_il1b_lps
pbmc_il10_cand
bmc_il1b_bbmix_10_
pbmc_il1b_p3c
wba_il6_bbmix_moi30
vba_il1b_bbmix_moi1
wba_il10_lps
wba_il6_p3c
bmc_il10_bbmix_10_
wba_il10_bbmix_moi3
pbmc_il1ra_cand
pbmc_il1ra_lps
pbmc_il1ra_p3c
pbmc_il10_p3c
bmc_il1ra_bbmix_10_
wba_il6_bbmix_moi10
wba_il6_bbmix_moi3
bmc_il1b_bbmix_10_
pbmc_il6_lps
pbmc_il6_p3c
vba_il10_bbmix_moi3
bmc_il10_bbmix_10_
vba_il10_bbmix_moi1
pbmc_il6_bbmix_10_5
pbmc_il6_bbmix_10_6
pbmc_il6_cand
pbmc_il6_bafzelii
pbmc_il10_bafzelii
bmc_il1ra_bbmix_10_
pbmc_il1ra_bafzelii
pbmc_il1ra_rpmi
wba_il1b_bbmix_moi3
bmc_il1ra_bbmix_10_
bmc_il1b_bbmix_10_
bmc_il1ra_bbmix_10_
pbmc_il1b_bafzelii
density
logvalue
Time
t0
t6
b
il10
IL-1β
IL-1Ra
il6
16
12
8
4
logvalue
t0
t6
Sample
pbmc
wba
bafzelii
bbmix moi10
bbmix moi3
bbmix moi30
bbmix 10 3
bbmix 10 4
bbmix 10 5
bbmix 10 6
C.albicans
LPS
Pam3Cys
RPMI
cand
Stimulation

Supplementary Figure 4. Distribution of log2 transformed Cytokine concentrations. a, Density plot of cytokine concentrations, color indicates the time point at which the sample was taken, red, at the start of antibiotic treatment, blue, after the completion of the antibiotic treatment. b, Boxplot of cytokine concentrations, colored by the cell system used, PBMCs or Whole Blood. Wba, Whole blood; cand, *Candida Albicans*; p3c, Pam3Cys; bbmix, *Borrelia Burgdorferi* mix; afzelii, *Borrelia Alfzelii;* t0, start of antibiotic treatment; t6, after antibiotic treatment. n=1,060.

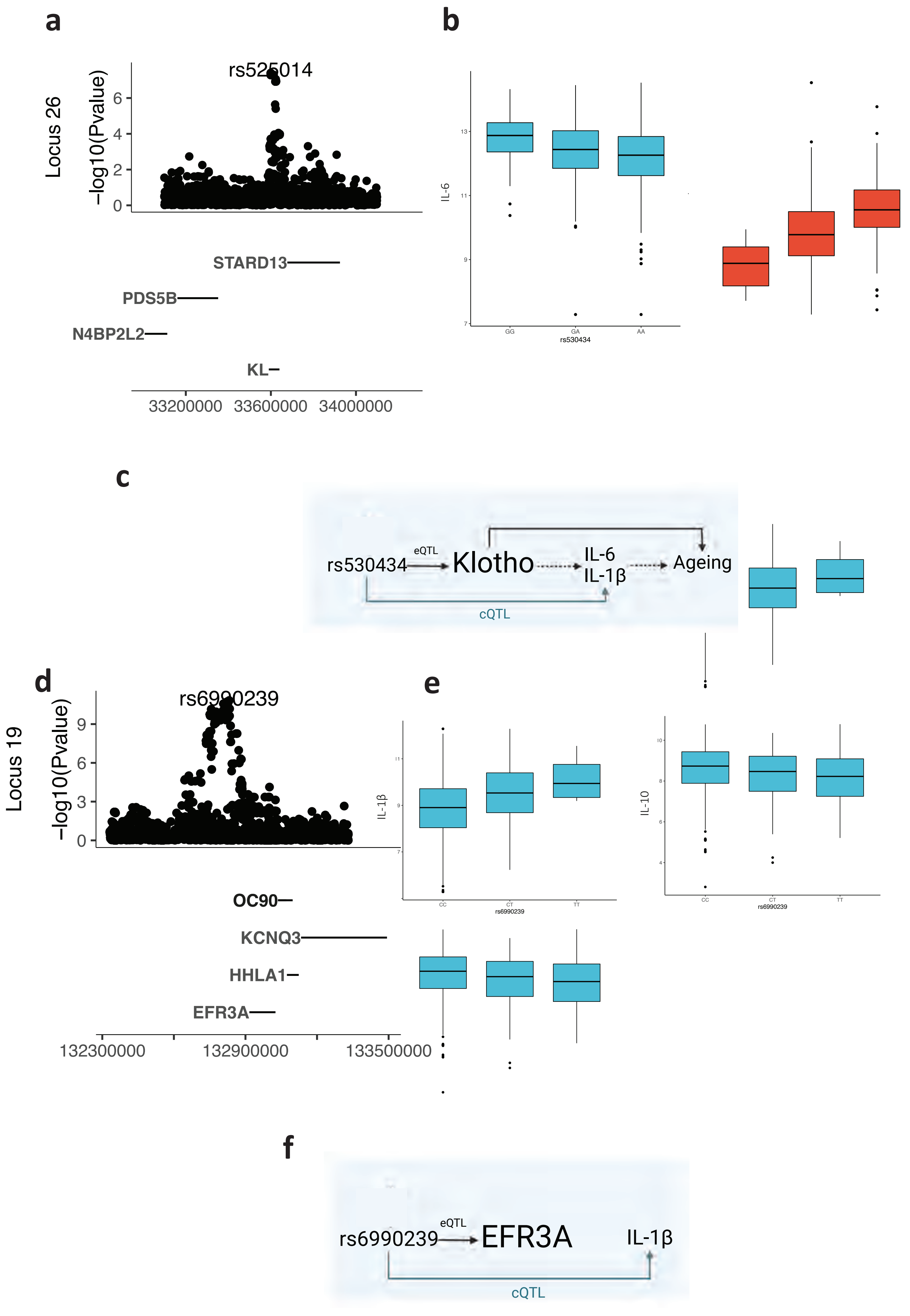
a
Locus 26
−log10(Pvalue)
rs525014
STARD13
PDS5B
N4BP2L2
KL
33200000
33600000
34000000
b
IL-6
rs530434
GG
GA
AA
c
rs530434
eQTL
Klotho
IL-6
IL-1β
Ageing
cQTL
d
Locus 19
−log10(Pvalue)
rs6990239
OC90
KCNQ3
HHLA1
EFR3A
132300000
132900000
133500000
e
IL-1β
IL-10
rs6990239
CC
CT
TT
f
rs6990239
eQTL
EFR3A
IL-1β
cQTL

Supplementary Figure 5. Two of the novel cQTL regulating cytokine responses: Klotho and *EFR3A*. a,d, Locuszoom plots of the top association. b,e Boxplot of the top associations. c,f, Schematic of the found and already known effects of the genomic loci. P-values are calculated based on a linear model associating cytokine concentrations with genetic variants and are shown without multiple testing correction n=1,060.

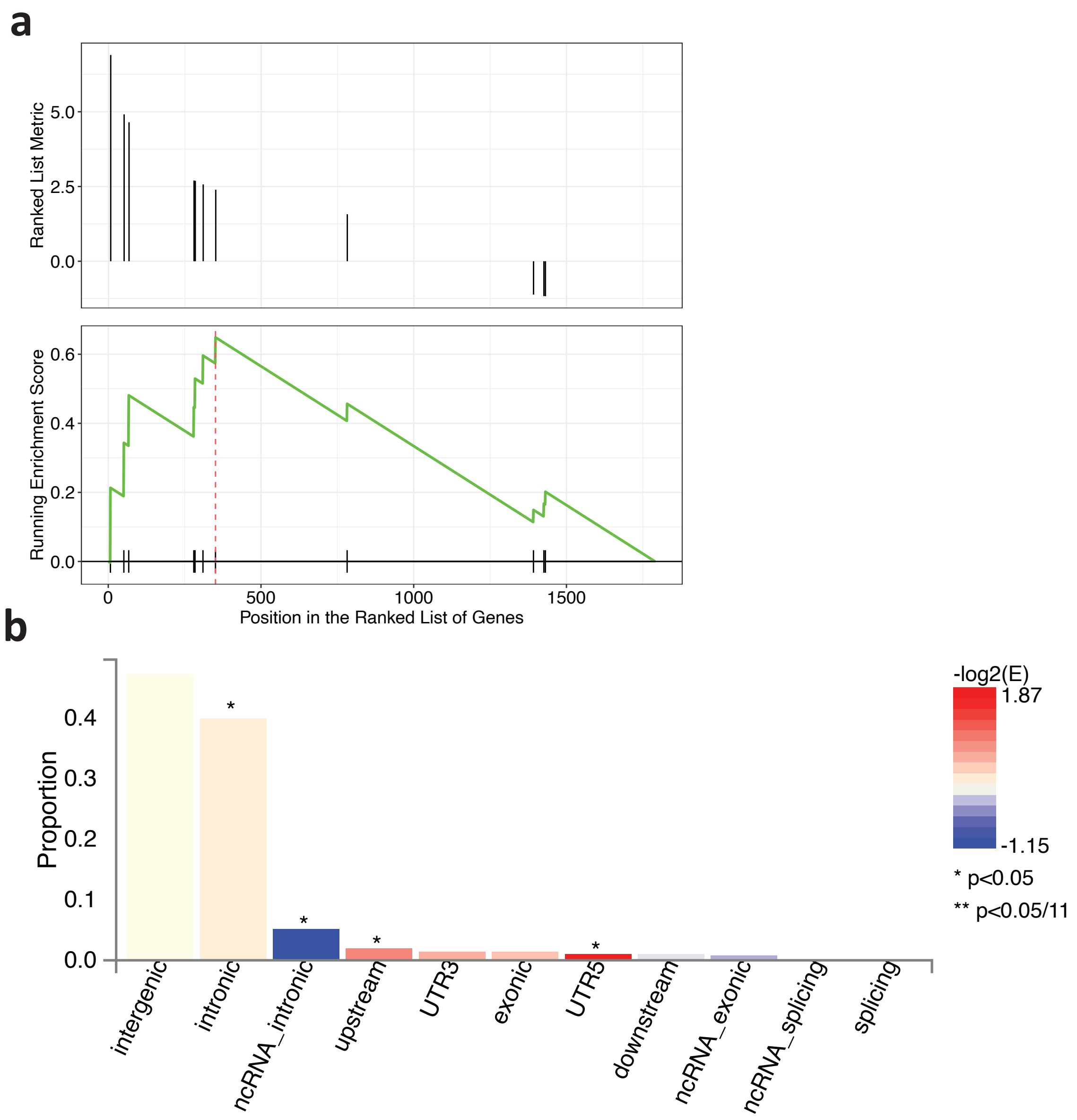
a
Ranked List Metric
5.0
2.5
0.0
Running Enrichment Score
0.6
0.4
0.2
0.0
0
500
1000
1500
Position in the Ranked List of Genes
b
Proportion
0.4
0.3
0.2
0.1
0.0
*
*
*
*
intergenic
intronic
ncRNA_intronic
upstream
UTR3
exonic
UTR5
downstream
ncRNA_exonic
ncRNA_splicing
splicing
-log2(E)
1.87
-1.15
* p<0.05
** p<0.05/11

Supplementary Figure 6. Enriched annotations in the identified cQTL. a, Enrichment score of *B. burgdorferi* cQTL genes in differentially-expressed genes upon *B. burgdorferi* stimulation. b, Genomic annotation of genome-wide cQTL SNPs. P-value estimated using Fisher's exact test between genome-wide significant QTLs and reference variants.

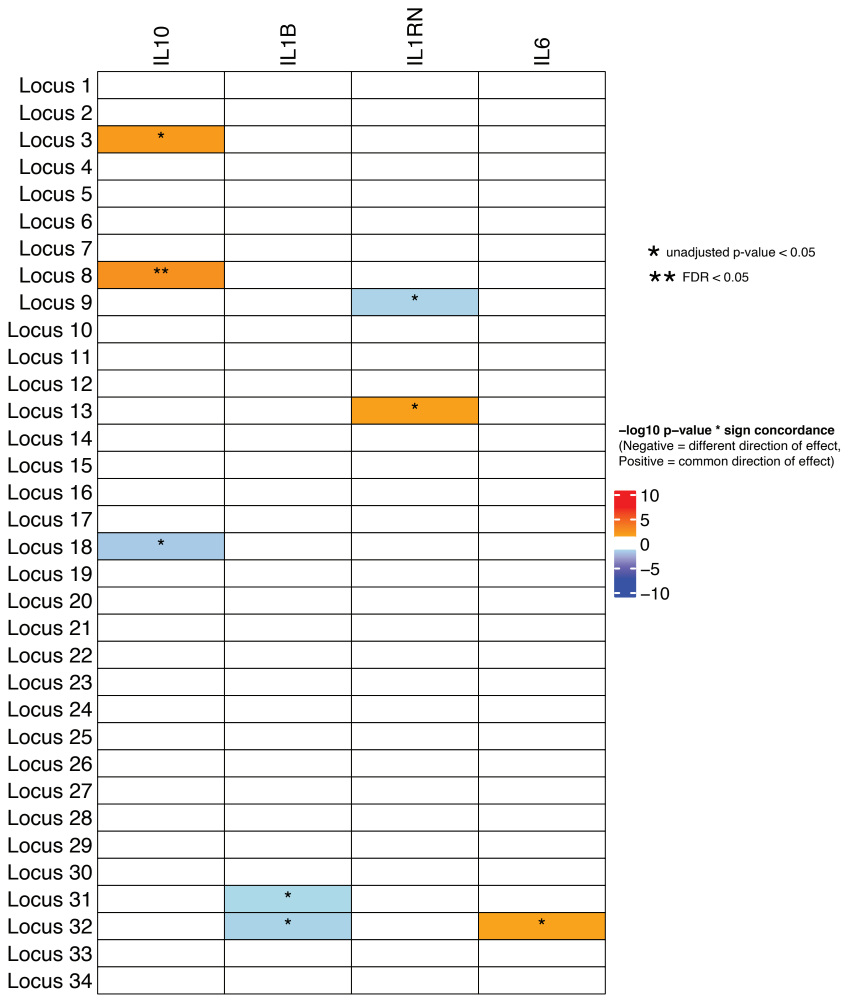
IL10
IL1B
IL1RN
IL6
Locus 1
Locus 2
Locus 3
Locus 4
Locus 5
Locus 6
Locus 7
Locus 8
Locus 9
Locus 10
Locus 11
Locus 12
Locus 13
Locus 14
Locus 15
Locus 16
Locus 17
Locus 18
Locus 19
Locus 20
Locus 21
Locus 22
Locus 23
Locus 24
Locus 25
Locus 26
Locus 27
Locus 28
Locus 29
Locus 30
Locus 31
Locus 32
Locus 33
Locus 34
* unadjusted p-value < 0.05
** FDR < 0.05
–log10 p–value * sign concordance
(Negative = different direction of effect,
Positive = common direction of effect)
10
5
0
–5
–10

Supplementary Figure 7. Replication of Genomewide cQTL in the UK biobank plasma protein QTL. See reference for methods. P-values are calculated based on a linear model associating cytokine concentrations with genetic variants and are shown without multiple testing correction n=1,060. * $P<0.05$ ** $FDR<0.05$ *** $P<5x10^{-8}$

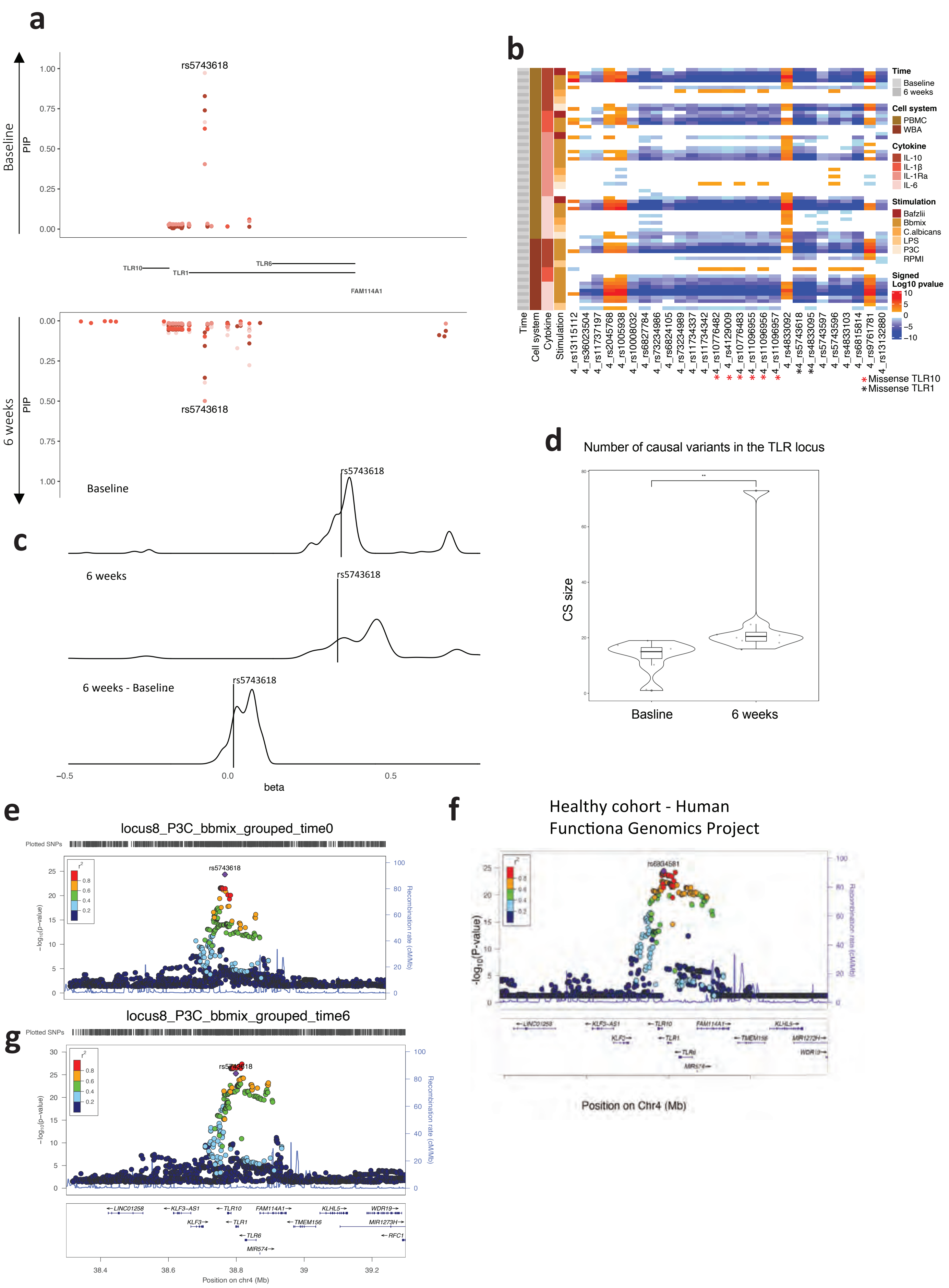
a
rs5743618
Baseline
PIP
1.00
0.75
0.50
0.25
0.00
TLR10
TLR1
TLR6
FAM114A1
6 weeks
b
Time
Baseline
6 weeks
Cell system
PBMC
WBA
Cytokine
IL-10
IL-1β
IL-1Ra
IL-6
Stimulation
Bafzlii
Bbmix
C.albicans
LPS
P3C
RPMI
Signed
Log10 pvalue
10
5
0
−5
−10
Time
Cell system
Cytokine
Stimulation
4_rs13115112
4_rs36023504
4_rs11737197
4_rs2045768
4_rs1005938
4_rs10008032
4_rs6827784
4_rs73234986
4_rs6824105
4_rs73234989
4_rs11734337
4_rs11734342
4_rs10776482
4_rs4129009
4_rs10776483
4_rs11096955
4_rs11096956
4_rs11096957
4_rs4833092
4_rs5743618
4_rs4833095
4_rs5743597
4_rs5743596
4_rs4833103
4_rs6815814
4_rs9761781
4_rs13132880
Missense TLR10
Missense TLR1
c
Baseline
6 weeks
6 weeks - Baseline
−0.5
0.0
0.5
beta
d
Number of causal variants in the TLR locus
CS size
Basline
6 weeks
e
locus8_P3C_bbmix_grouped_time0
Plotted SNPs
−log10(p-value)
Recombination rate (cM/Mb)
f
Healthy cohort - Human
Functiona Genomics Project
rs6834581
Position on Chr4 (Mb)
g
locus8_P3C_bbmix_grouped_time6
LINC01258
KLF3−AS1
TLR10
FAM114A1
KLHL5
WDR19
KLF3
TLR1
TMEM156
MIR1273H
TLR6
RFC1
MIR574
38.4
38.6
38.8
39
39.2
Position on chr4 (Mb)

Supplementary Figure 8. Exploring further the *TLR1-6-10* locus. a, Fine mapping posterior probability (PIP) of top fine-mapped SNPs in the TLR locus for two different TLR2 ligands, colored by the cytokine measured. b, Heatmap of association significance of the top independent variants in the TLR locus. c, Distribution of effect sizes (beta) of cQTL associations at Baseline, After antibiotic treatment and the subtraction of baseline effects from the six weeks' time point. d, Credible-set size of TLR locus fine-mapping for all conditions measured, separated by the time of extraction of the sample. e-g Locuszoom of stimulation-grouped cQTL associations at baseline, e, after treatment, g, and figure extracted from the study in healthy individuals (Li et al., 2016, Cell).

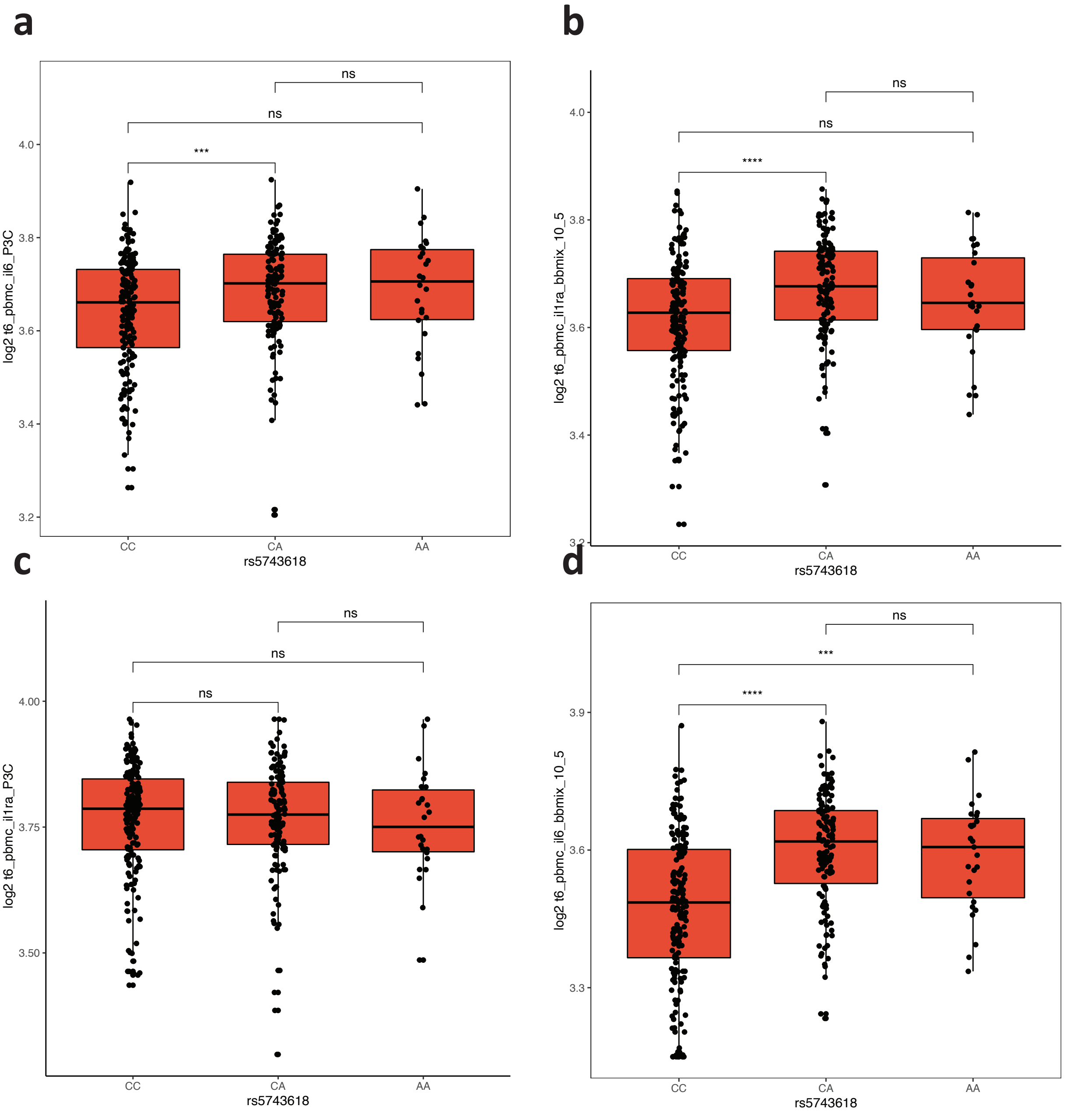
a
ns
ns
***
4.0
3.8
3.6
3.4
3.2
log2 t6_pbmc_il6_P3C
CC
CA
AA
rs5743618
b
ns
ns
****
4.0
3.8
3.6
3.4
3.2
log2 t6_pbmc_il1ra_bbmix_10_5
CC
CA
AA
rs5743618
c
ns
ns
ns
4.00
3.75
3.50
log2 t6_pbmc_il1ra_P3C
CC
CA
AA
rs5743618
d
ns
***
****
3.9
3.6
3.3
log2 t6_pbmc_il6_bbmix_10_5
CC
CA
AA
rs5743618

Supplementary Figure 9. Differences in the regulation of Pam3Cys and *B. burgdorferi* responses by the *TLR1-6-10* locus, data after treatment. a-d, IL-6 responses to Pam3Cys, a, *B. burgdorferi* at 10^5, d, IL-1Ra responses to Pam3Cys, c, and *B. burgdorferi* at 10^5, b. P-values calculated with a two-sided Wilcoxon rank-sum test (n=1,060), * P<0.05 ** P<0.01 *** P<0.001 *** P<0.0001.

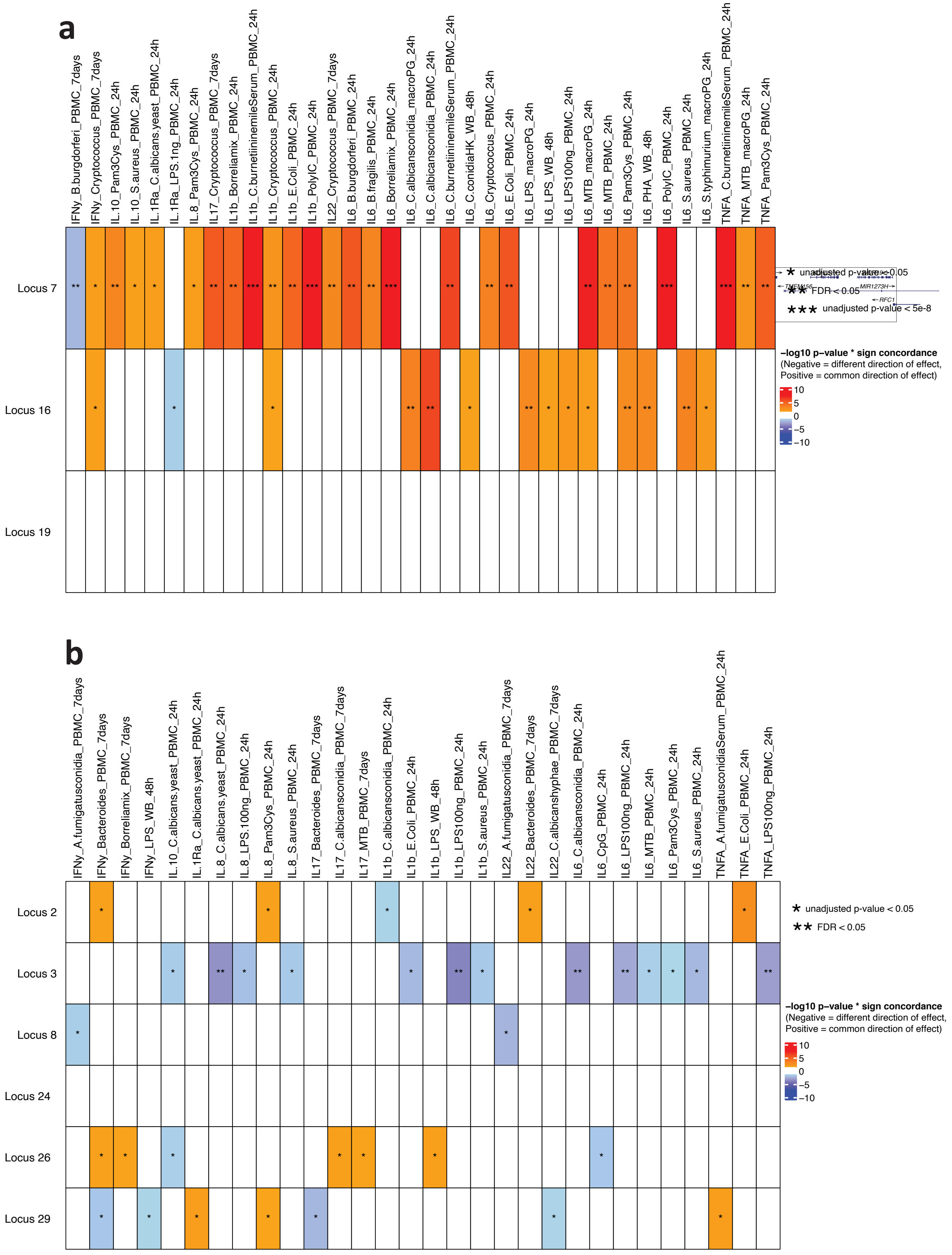

a
IFNy_B.burgdorferi_PBMC_7days
IFNy_Cryptococcus_PBMC_7days
IL.10_Pam3Cys_PBMC_24h
IL.10_S.aureus_PBMC_24h
IL.1Ra_C.albicans.yeast_PBMC_24h
IL.1Ra_LPS.1ng_PBMC_24h
IL.8_Pam3Cys_PBMC_24h
IL17_Cryptococcus_PBMC_7days
IL1b_Borreliamix_PBMC_24h
IL1b_C.burnetiininemileSerum_PBMC_24h
IL1b_Cryptococcus_PBMC_24h
IL1b_E.Coli_PBMC_24h
IL1b_PolyIC_PBMC_24h
IL22_Cryptococcus_PBMC_7days
IL6_B.burgdorferi_PBMC_24h
IL6_B.fragilis_PBMC_24h
IL6_Borreliamix_PBMC_24h
IL6_C.albicansconidia_macroPG_24h
IL6_C.albicansconidia_PBMC_24h
IL6_C.burnetiininemileSerum_PBMC_24h
IL6_C.conidiaHK_WB_48h
IL6_Cryptococcus_PBMC_24h
IL6_E.Coli_PBMC_24h
IL6_LPS_macroPG_24h
IL6_LPS_WB_48h
IL6_LPS100ng_PBMC_24h
IL6_MTB_macroPG_24h
IL6_MTB_PBMC_24h
IL6_Pam3Cys_PBMC_24h
IL6_PHA_WB_48h
IL6_PolyIC_PBMC_24h
IL6_S.aureus_PBMC_24h
IL6_S.typhimurium_macroPG_24h
TNFA_C.burnetiininemileSerum_PBMC_24h
TNFA_MTB_macroPG_24h
TNFA_Pam3Cys_PBMC_24h
Locus 7
Locus 16
Locus 19
* unadjusted p-value < 0.05
** FDR < 0.05
*** unadjusted p-value < 5e-8
TMEM156
MIR1273H
RFC1
–log10 p–value * sign concordance
(Negative = different direction of effect,
Positive = common direction of effect)
10
5
0
−5
−10
b
IFNy_A.fumigatusconidia_PBMC_7days
IFNy_Bacteroides_PBMC_7days
IFNy_Borreliamix_PBMC_7days
IFNy_LPS_WB_48h
IL.10_C.albicans.yeast_PBMC_24h
IL.1Ra_C.albicans.yeast_PBMC_24h
IL.8_C.albicans.yeast_PBMC_24h
IL.8_LPS.100ng_PBMC_24h
IL.8_Pam3Cys_PBMC_24h
IL.8_S.aureus_PBMC_24h
IL17_Bacteroides_PBMC_7days
IL17_C.albicansconidia_PBMC_7days
IL17_MTB_PBMC_7days
IL1b_C.albicansconidia_PBMC_24h
IL1b_E.Coli_PBMC_24h
IL1b_LPS_WB_48h
IL1b_LPS100ng_PBMC_24h
IL1b_S.aureus_PBMC_24h
IL22_A.fumigatusconidia_PBMC_7days
IL22_Bacteroides_PBMC_7days
IL22_C.albicanshyphae_PBMC_7days
IL6_C.albicansconidia_PBMC_24h
IL6_CpG_PBMC_24h
IL6_LPS100ng_PBMC_24h
IL6_MTB_PBMC_24h
IL6_Pam3Cys_PBMC_24h
IL6_S.aureus_PBMC_24h
TNFA_A.fumigatusconidiaSerum_PBMC_24h
TNFA_E.Coli_PBMC_24h
TNFA_LPS100ng_PBMC_24h
Locus 2
Locus 3
Locus 8
Locus 24
Locus 26
Locus 29
* unadjusted p-value < 0.05
** FDR < 0.05
–log10 p–value * sign concordance
(Negative = different direction of effect,
Positive = common direction of effect)
10
5
0
−5
−10

Supplementary Figure 10. Replication of cQTL in a healthy cohort. Summary statistics extracted from Li et al., Cell, 2016. See reference for methods. * $P<0.05$ ** $FDR<0.05$ *** $P<5x10^{-8}$. a, Study-wide significant loci, b, Genome-wide significant loci. P-values are calculated based on a linear model associating cytokine concentrations with genetic variants and are shown without multiple testing correction n=1,060.

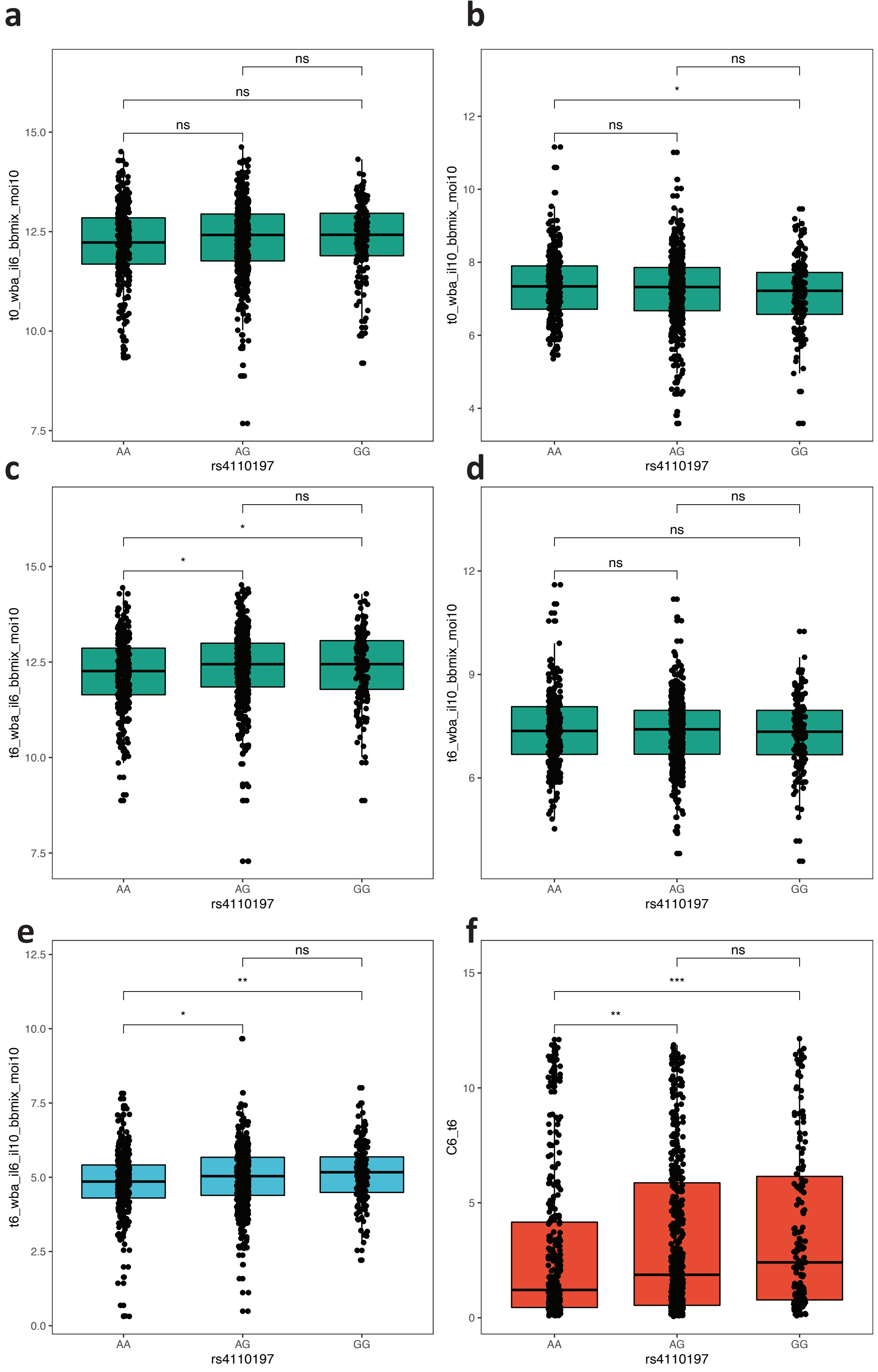
a
b
c
d
e
f
ns
*
**
***
t0_wba_il6_bbmix_moi10
t0_wba_il10_bbmix_moi10
t6_wba_il6_bbmix_moi10
t6_wba_il10_bbmix_moi10
t6_wba_il6_il10_bbmix_moi10
C6_t6
AA
AG
GG
rs4110197

Supplementary Figure 11. Effects of the Finngen Lyme susceptibility variant on cytokine production in *B. burgdorferi* stimulated whole blood. Baseline (a,b) and after antibiotic treatment (c,d) effect of the susceptibility variant on IL-6 (a, c) and IL-10 (b, d) upon *B. burgdorferi* stimulation on whole blood. e, After treatment ratio of IL-6 and IL-10 concentrations. f, After treatment effect on C6 antibody index (IgG/IgM) levels. P-values calculated with a two-sided Wilcoxon rank-sum test, n=1,060, * P<0.05 ** P<0.01 *** P<0.001 *** P<0.0001.

a

IL-6 production upon *Bb* stimulation of PBMCs at the 6 weeks timepoint

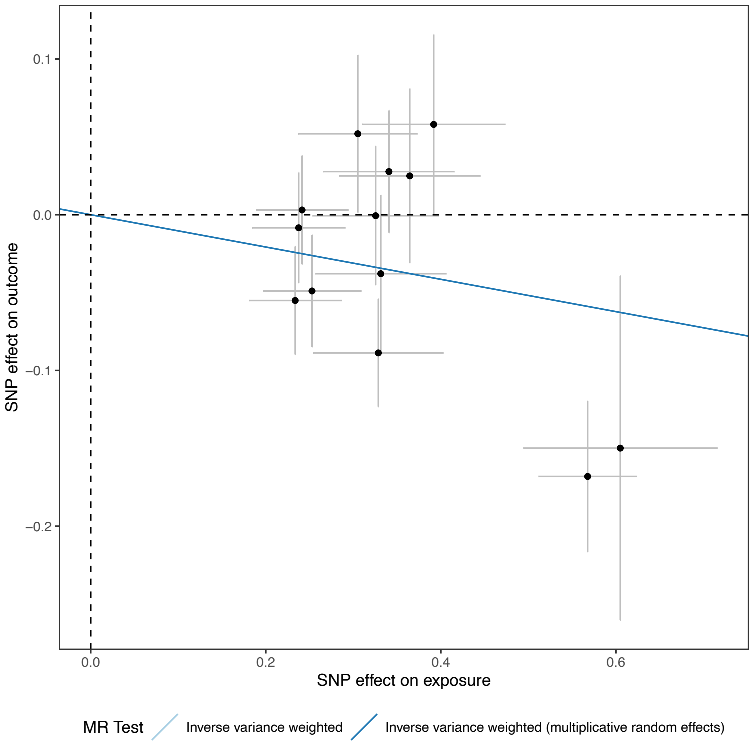

b

IL-10 production upon *Bb* stimulation of PBMCs at the baseline

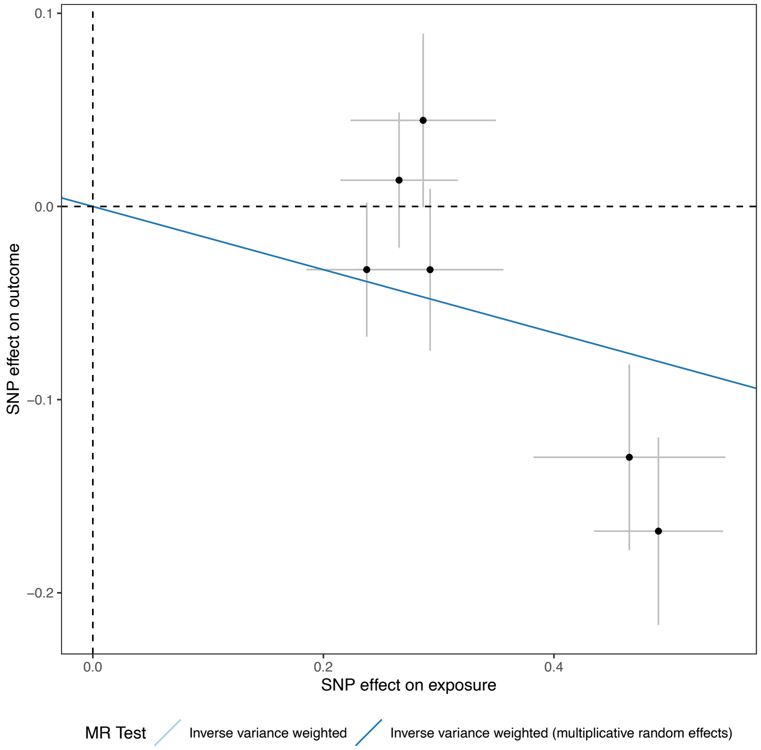

Supplementary Figure 12. Mendelian randomization of cytokine production capacity upon *B. burgdorferi* stimulation in PBMCs. Exposures**:** a, IL-6 response upon *B. burgdorferi* stimulation in PBMCs after antibiotic treatment. b, IL-10 in *B. burgdorferi* stimulated PBMCs at baseline. Dark blue like, multiplicative random effects inverse variance weighted meta-analysis.

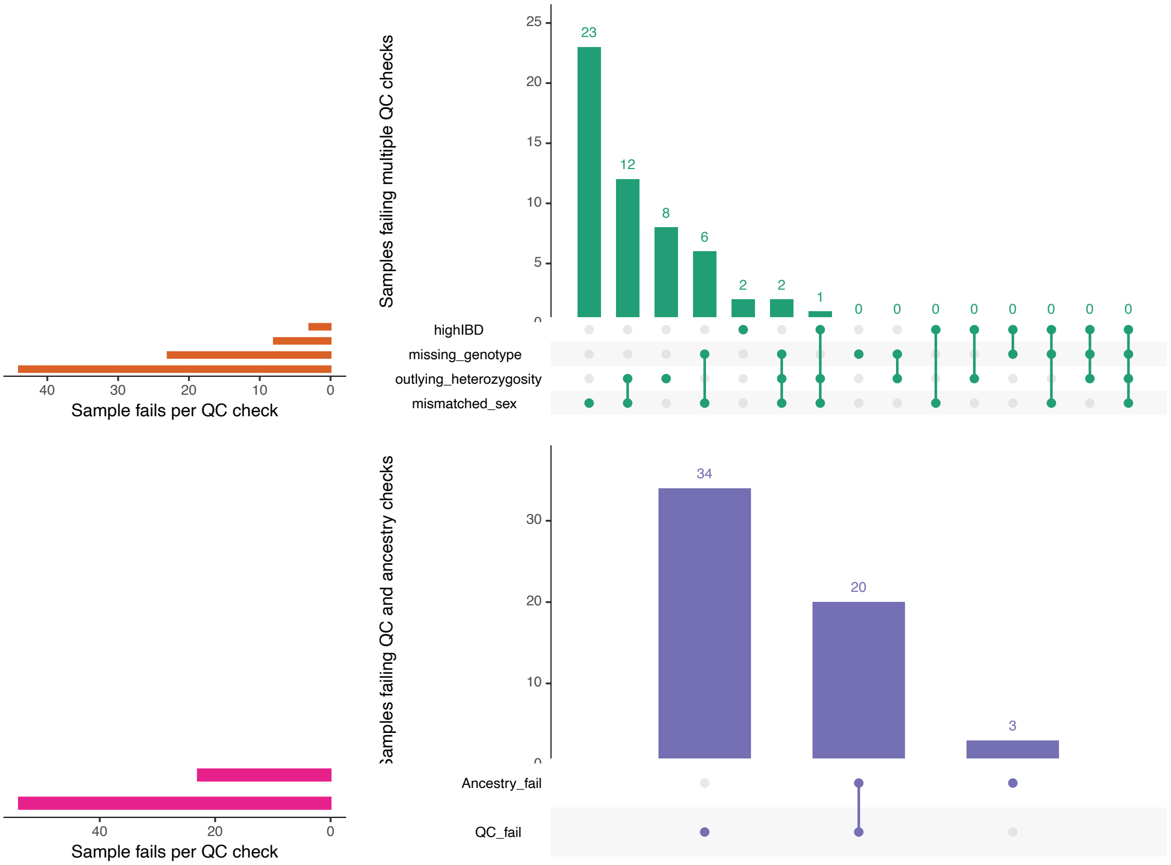
Samples failing multiple QC checks
highIBD
missing_genotype
outlying_heterozygosity
mismatched_sex
Sample fails per QC check
23
12
8
6
2
2
1
0
amples failing QC and ancestry checks
34
20
3
Ancestry_fail
QC_fail
Sample fails per QC check

Supplementary Figure 13. Quality-control of genotype samples. Top plot, general QC of relatedness, missingness, heterozygosity and sex mismatch. Bottom plot, summary of general QC and ancestry check.
