# Supplementary material for: A comprehensive genetic map of cytokine responses in Lyme borreliosis
Source: Nat Commun. 2024 May 7;15:3795. doi: 10.1038/s41467-024-47505-z (PMC11076587; doi:10.1038/s41467-024-47505-z)
Supplement: Supplementary file 3 — Description of Additional Supplementary Files [file 41467_2024_47505_MOESM3_ESM.pdf]

Description of Additional Supplementary Files

File Name: Supplementary Data 1
Description: Information on whether the patient had started the antibiotic treatment at the moment of sample collection.

File Name: Supplementary Data 2
Description: Covariates and clinical measurements taken from all participants. Information on age, biological sex, diameter of the EM lesion and C6 ratios at baseline and 6 weeks after. Cytokine concentrations upon inactivated pathogen stimulation, raw concentrations.

File Name: Supplementary Data 3
Description: Results of the interaction analysis between the top TLR1 variant and both TLR2 stimulation. Linear model with interaction applied.

File Name: Supplementary Data 4
Description: Results of the differential expression analyses between the two different alleles for rs5743618 and gene expression in the validation cohort of 100 Healthy individuals.

File Name: Supplementary Data 5
Description: GWAS summary statistics from the GWASCatalog used for the colocalication analysis with cytokine QTLs in Lyme Borreliosis.

File Name: Supplementary Data 6
Description: Results from single-snp mendelian randomization between significantly colocalized cytokine QTLs and immune-mediated diseases.

File Name: Supplementary Data 7
Description: Results of the colocalization analysis between Lyme susceptibility in the Finngen cohort and cytokine QTL in the LymeProspect cohort.

File Name: Supplementary Data 8
Description: Results of the colocalization analysis between Lyme susceptibility in the Finngen cohort and cytokine QTL in the LymeProspect cohort.

File Name: Supplementary Data 9
Description: Results of the mendelian randomisation analysis between Lyme susceptibility in the Finngen cohort and cytokine QTL in the LymeProspect cohort.
