# Supplementary material for: A comprehensive genetic map of cytokine responses in Lyme borreliosis
Source: Nat Commun. 2024 May 7;15:3795. doi: 10.1038/s41467-024-47505-z (PMC11076587; doi:10.1038/s41467-024-47505-z)
Supplement: Supplementary file 7 — Reporting Summary [file 41467_2024_47505_MOESM7_ESM.pdf]

Corresponding author(s): Yang Li, Leo A.B. Joosten

Last updated by author(s): Sep 7, 2023

Reporting Summary

Nature Portfolio wishes to improve the reproducibility of the work that we publish. This form provides structure for consistency and transparency in reporting. For further information on Nature Portfolio policies, see our Editorial Policies and the Editorial Policy Checklist.

Statistics

For all statistical analyses, confirm that the following items are present in the figure legend, table legend, main text, or Methods section.

n/a	Confirmed	
☐	☒	The exact sample size (*n*) for each experimental group/condition, given as a discrete number and unit of measurement
☐	☒	A statement on whether measurements were taken from distinct samples or whether the same sample was measured repeatedly
☐	☒	The statistical test(s) used AND whether they are one- or two-sided<br>*Only common tests should be described solely by name; describe more complex techniques in the Methods section.*
☐	☒	A description of all covariates tested
☐	☒	A description of any assumptions or corrections, such as tests of normality and adjustment for multiple comparisons
☐	☒	A full description of the statistical parameters including central tendency (e.g. means) or other basic estimates (e.g. regression coefficient) AND variation (e.g. standard deviation) or associated estimates of uncertainty (e.g. confidence intervals)
☐	☒	For null hypothesis testing, the test statistic (e.g. *F*, *t*, *r*) with confidence intervals, effect sizes, degrees of freedom and *P* value noted<br>*Give P values as exact values whenever suitable.*
☒	☐	For Bayesian analysis, information on the choice of priors and Markov chain Monte Carlo settings
☒	☐	For hierarchical and complex designs, identification of the appropriate level for tests and full reporting of outcomes
☐	☒	Estimates of effect sizes (e.g. Cohen's *d*, Pearson's *r*), indicating how they were calculated

Our web collection on statistics for biologists contains articles on many of the points above.

Software and code

Policy information about availability of computer code

Data collection	GenomeStudio v.2011.11
Data analysis	R 4.0.1, PLINK v1.90b6.12, PLINK v2.00a3LM, FUMA used on 08/2022, Michigan Imputation Server used on 08/2022. R packages: MatrixEQTL 2.3, tidyverse 1.3.1, data.table 1.14.2, plinkQC 0.3.4, coloc 5.1.0.1, susieR 0.12.16, ggpubr 0.6.0, ComplexHeatmap 2.12.0, TwoSampleMR 0.5.6.<br>Custom-generated scripts are deposited on Github (https://github.com/CiiM-Bioinformatics-group/Lyme_cQTL)

For manuscripts utilizing custom algorithms or software that are central to the research but not yet described in published literature, software must be made available to editors and reviewers. We strongly encourage code deposition in a community repository (e.g. GitHub). See the Nature Portfolio guidelines for submitting code & software for further information.

Data

Policy information about availability of data

All manuscripts must include a data availability statement. This statement should provide the following information, where applicable:

- Accession codes, unique identifiers, or web links for publicly available datasets
- A description of any restrictions on data availability
- For clinical datasets or third party data, please ensure that the statement adheres to our policy

Cytokine expression and C6 antibody ratios are attached as a supplementary table. Summary statistics are available on the GWAS Catalog (study ids GCST90275731

to GCST90275790). Genotype array data is available through EGA at EGAS50000000024. Significant cytokine QTL results are available at https://lab-li.ciim-hannover.de/apps/lyme_cqtl/.

Research involving human participants, their data, or biological material

Policy information about studies with human participants or human data. See also policy information about sex, gender (identity/presentation), and sexual orientation and race, ethnicity and racism.

Reporting on sex and gender	The study included patients of both sexes. Sexes were self-reported and confirmed by DNA genotyping. For association analyses, sex was included as a covariate. No sex-specific analyses were performed as this was not part of the scope of the manuscript.
Reporting on race, ethnicity, or other socially relevant groupings	All study participants were of self-reported European ancestry that was later confirmed with the DNA genotyping data. No other genetic ancestries were included in order to increase the statistical power to detect associations. No specific socially constructed variables were used.
Population characteristics	All study participants had been diagnosed for Lyme Borreliosis and the diagnosis was confirmed by a Physician. All participants were of European ancestry. Participants' age were between 19 and 87 years old, with a median of 55. 41% (435) of the participants were male. 96% (1,017) of the participants presented a localized infection (Erythrema migrans), the rest presented a disseminated infection. 87% of the participants had started with an antibiotic treatment at the first time point of sample collection. All participants characteristics are summarized in Extended Data Table 1.
Recruitment	Patients were included after online self-registration (www.tekenradar.nl) or through participating clinical LB centers. For patients with EM included after online self-registration, their general practitioner was asked to confirm the diagnosis. Patients were requested to upload a photograph of their EM, which was classified by three independent experts (one dermatologist and two infectious diseases specialists). All patients were included before, or within a maximum of seven days after the start of antibiotic treatment. Inclusion did not interfere with standard diagnostic and therapeutic procedures.
Ethics oversight	The study was approved by the medical ethics review committee Noord-Holland (NL50227.094.14) and conducted in accordance with the Declaration of Helsinki.

Note that full information on the approval of the study protocol must also be provided in the manuscript.

Field-specific reporting

Please select the one below that is the best fit for your research. If you are not sure, read the appropriate sections before making your selection.

☒ Life sciences ☐ Behavioural & social sciences ☐ Ecological, evolutionary & environmental sciences

For a reference copy of the document with all sections, see nature.com/documents/nr-reporting-summary-flat.pdf

Life sciences study design

All studies must disclose on these points even when the disclosure is negative.

Sample size	No statistical tests were undertaken to determine the sample size a priori, and sample sizes were limited by clinical availability. Details on sample availability are detailed in Ursinus et al., The Lancet Regional Health 2021.
Data exclusions	Inclusion criteria<br>1. Patients ≥18 years with confirmed proven or probable early localised or disseminated LB manifestation as specified below;<br>2. In case of an EM reported online (at www.tekenradar.nl), the EM has been present <3 months and the clinical diagnosis has been confirmed to the research staff by the general practitioner (criteria for clinical diagnosis are described below);<br>3. Subjects live or stay on the mainland of the Netherlands.<br>Exclusion criteria<br>1. Subjects unable to provide informed consent or not having sufficient command of the Dutch language;<br>2. Subjects who started antibiotic treatment >4 days before inclusion (for subjects included after online reporting) or >7 days before inclusion (for subjects included through the participating clinical LB centers);<br>3. Subjects who have ongoing signs or symptoms attributed to a previous episode of LB
Replication	All cytokine QTL results were tested for replication in an independent cohort of healthy individuals, confirming a modest replication in healthy, in which 7 of the 9 identified loci were replicated ($P<0.05$).<br>All cytokine QTLs were tested for replication in an independent pQTL study.<br>The association between rs5743618 and two cytokines responses was tested at gene expression level in an independent cohort of healthy individuals.
Randomization	This study included patients with Lyme Borreliosis. Since the main scope of the analyses was to test the association between common genetic variants and immune phenotypes, no randomization was performed. Study participants were balanced in age (median 53.5) and biological sex (59% female participants). Age and sex were included as covariates in the linear models that tested the association between cytokine responses and genetic variants.
Blinding	All experiments were conducted in an unblinded way since the investigators were involved in the planning, execution and analyses of the

Blinding	current study.

Reporting for specific materials, systems and methods

We require information from authors about some types of materials, experimental systems and methods used in many studies. Here, indicate whether each material, system or method listed is relevant to your study. If you are not sure if a list item applies to your research, read the appropriate section before selecting a response.

Materials & experimental systems

n/a	Involved in the study
☒	☐ Antibodies
☒	☐ Eukaryotic cell lines
☒	☐ Palaeontology and archaeology
☒	☐ Animals and other organisms
☒	☐ Clinical data
☒	☐ Dual use research of concern
☒	☐ Plants

Methods

n/a	Involved in the study
☒	☐ ChIP-seq
☒	☐ Flow cytometry
☒	☐ MRI-based neuroimaging
